# Identification and quantification of glucosinolate and flavonol compounds in rocket salad (*Eruca sativa*, *Eruca vesicaria* and *Diplotaxis tenuifolia*) by LC–MS: Highlighting the potential for improving nutritional value of rocket crops

**DOI:** 10.1016/j.foodchem.2014.09.116

**Published:** 2015-04-01

**Authors:** Luke Bell, Maria Jose Oruna-Concha, Carol Wagstaff

**Affiliations:** a Department of Food & Nutritional Sciences, University of Reading, PO Box 226, Whiteknights, Reading, Berkshire RG6 6AP, UK; b Centre for Food Security, University of Reading, Whiteknights, Reading, Berkshire RG6 6AH, UK

**Keywords:** Glucoerucin (PubChem CID: 656539), Glucoraphanin (PubChem CID: 9548633), 4-Hydroxyglucobrassicin (PubChem CID: 49859657), Glucolepiidin (PubChem CID: 656547), Glucoiberverin (PubChem CID: 9548637), Glucoalyssin (PubChem CID: 9589398), Glucoraphenin (PubChem CID: 9548613), Glucoibarin (PubChem CID: 44237203), Astragalin (PubChem CID: 5282102), Isoquercetrin (PubChem CID: 5280804), *Brassicaceae*, Phytochemicals, Liquid chromatography mass spectrometry, Controlled environment, Plant breeding, Gene bank

## Abstract

Liquid chromatography mass spectrometry (LC–MS) was used to obtain glucosinolate and flavonol content for 35 rocket accessions and commercial varieties. 13 glucosinolates and 11 flavonol compounds were identified. Semi-quantitative methods were used to estimate concentrations of both groups of compounds. Minor glucosinolate composition was found to be different between accessions; concentrations varied significantly. Flavonols showed differentiation between genera, with *Diplotaxis* accumulating quercetin glucosides and *Eruca* accumulating kaempferol glucosides. Several compounds were detected in each genus that have only previously been reported in the other. We highlight how knowledge of phytochemical content and concentration can be used to breed new, nutritionally superior varieties. We also demonstrate the effects of controlled environment conditions on the accumulations of glucosinolates and flavonols and explore the reasons for differences with previous studies. We stress the importance of consistent experimental design between research groups to effectively compare and contrast results.

## Highlights

• 13 glucosinolate and 11 flavonol compounds identified across 35 rocket accessions.
• Several flavonol compounds are newly identified in *Eruca* and *Diplotaxis* species.
• Commercial varieties show enhanced flavonol concentrations but low glucosinolates.
• We stress the importance of consistent and commercially focused experimental design.

## 1 Introduction

The groups of crops collectively known as rocket (or arugula, rucola, roquette) are all members of the *Brassicaceae* family, and are native to the areas surrounding the Mediterranean Sea (Martinez-Sanchez et al., 2006). Rocket crops belong to two genera, *Eruca* and *Diplotaxis*, and are increasingly important in the salad vegetable market (Pasini, Verardo, Cerretani, Caboni, & D’Antuono, 2011). The species are now grown commercially all over the world in countries as diverse as the USA, UK, Italy, Spain, Morocco, Israel, India and Australia (Bozokalfa, Esiyok, & Yagmur, 2011).

Previous studies have highlighted rocket as a rich source of glucosinolate (GSL) compounds (Kim, Jin, & Ishii, 2004). Virtually all other members of the *Brassicaceae* contain GSLs as secondary metabolites that act as part of plant defence mechanisms (Schranz, Manzaneda, Windsor, Clauss, & Mitchell-Olds, 2009). GSLs and their hydrolysis products have also been implicated in giving rocket its characteristic pungent aromas and flavours (Bennett et al., 2002) and volatiles (such as isothiocyanates (ITCs) and indoles) have been consistently linked with anticarcinogenic activity in mammalian tissues (Lynn, Collins, Fuller, Hillman, & Ratcliffe, 2006).

Both *Eruca* and *Diplotaxis* species contain similar profiles of GSLs within the leaf tissue, the most prominent of which are glucosativin (4-mercaptobutyl-GSL), glucoerucin (4-(methylthio)butyl-GSL) and glucoraphanin (4-(methylsulfinyl)butyl-GSL). Glucosativin and glucoerucin breakdown products are thought to contribute most to pungency and flavour in rocket (Pasini, Verardo, Caboni, & D’Antuono, 2012). Numerous other GSLs have also been identified within rocket tissue, for example diglucothiobeinin (4-(β-D-glucopyranosyldisulfanyl)butyl-GSL) (Kim et al., 2007), 4-hydroxyglucobrassicin (4-hydroxy-3-indolymethyl-GSL) (Cataldi, Rubino, Lelario, & Bufo, 2007) and 4-methoxyglucobrassicin (4-methoxy-3-indolymethyl-GSL) (Kim & Ishii, 2006).

Rocket species also contain large concentrations of polyglycosylated flavonol compounds, which are known to infer numerous beneficial health effects in humans and other animals. Particularly of note are their effects on the gastrointestinal tract and in cardiovascular health (Bjorkman et al., 2011, Traka and Mithen, 2011). Several studies in rocket have identified and quantified polyglycosylated flavonols, which belong to three core aglycones: isorhamnetin, kaempferol and quercetin (Bennett, Rosa, Mellon, & Kroon, 2006).

Prolonged intake of *Brassicaceae* vegetables and leaves has a demonstrably beneficial impact on human health (D’Antuono et al., 2009); however much of the world’s population do not consume enough of them to have these benefits, as is highlighted in several studies (Casagrande, Wang, Anderson, & Gary, 2007). Therefore, instead of only promoting increased consumption of leafy vegetables such as rocket, we propose increasing the nutritional quality and phytochemical density of varieties by using advanced screening and plant breeding methods, whilst still maintaining the sensory and visual acceptance of the consumer. This has already been achieved in broccoli with the production of varieties such as Beneforte which accumulates high concentrations of glucoraphanin (Traka et al., 2013).

In this study we draw a comparison between commercial rocket varieties available for public consumption and underutilised genetic resources. Nineteen gene bank accessions of *Eruca sativa* and sixteen commercial varieties (comprising *E. sativa*, *Eruca vesicaria* and *Diplotaxis tenuifolia*) were evaluated for GSL and polyglycosylated flavonol composition under controlled environment conditions. We hypothesise that through selective breeding for morphological traits in rocket, many important health promoting phytochemical traits may have been lost in commercial varieties, and that by breeding from underutilised accessions, nutritionally superior varieties can be produced. We also hypothesise that controlled environment growing conditions minimizes the effects of environmental stress on rocket plants, and provides a platform for comparable results between research groups and repeat experiments. We also call on other groups to consider plant maturity and time of harvest as an important factor in determining the usefulness of data to breeders and growers.

## 2 Materials and methods

### 2.1 Plant material

Rocket accessions were selected from three European gene banks based upon information provided by Elsoms Seeds Ltd. (Spalding, Lincolnshire, UK). In total 19 were sourced; 2 from the Centre for Genetic Resources in the Netherlands (CGN, Wageningen, The Netherlands), 12 from the Leibniz-Institut für Pflanzengenetik und Kulturpflanzenforschung (IPK, Gatersleben, Germany), and 5 from the University of Warwick Crop Centre Genetic Resources Unit (Wellesbourne, UK; formerly Warwick HRI). A further 16 commercial varieties were collected: 13 were independently sourced from retailers, 1 provided by Elsoms Seeds Ltd., and 2 from Bakkavor Group Ltd. (Bourne, Lincolnshire, UK).

Three biological replicates of each accession/variety were germinated under controlled environmental conditions (in Saxcil growth cabinets) after being sown in a random sequence. Long-day lighting was used (16 h light, 8 h dark) at an intensity of 200 μmol m^−2^ s^−1^ (equivalent to 10,800 Lux of sunlight). Daytime temperatures were set at 20 °C and nighttime temperatures at 14 °C. Seedlings were grown for ten days in seedling trays and then transplanted to larger trays; four plants of each replicate were grown on. Plants were grown for another twenty days and then leaves from the four plants were harvested together. Sampling for each plant took approximately one minute from the cutting of the leaves at the petiole to being placed in zip-loc freezer bags on dry ice inside a polystyrene container (with lid). For health and safety reasons it was decided that liquid nitrogen would not be used in this process.

Thirty days was chosen as the optimum point of harvest as it reflects the typical number of days commercial growers grow their crop after sowing. Bags were placed in a −80 °C freezer immediately after harvest and transport was completed (<30 min). Samples were freeze-dried in batches for three days (in a Vertis Bench-top Series). Leaves from each rep were ground into a fine powder using a combination of pestle and mortar and miniature coffee grinder (De’Longhi KG49, Treviso, Italy).

### 2.2 Reagents and chemicals

All solvents and chemicals used were of LC–MS grade and obtained from Sigma–Aldrich (Poole, UK) unless otherwise stated.

### 2.3 Glucosinolate/flavonol extraction

The following method was adapted from Pasini, Verardo, Caboni, and D’Antuono (2012), Jin et al. (2009). Three experimental replicates of each biological rep were prepared as follows: 40 mg of ground rocket powder was heated in a dry-block at 75 °C for 2 min, as suggested by Pasini, Verardo, Caboni, and D’Antuono (2012), as a precautionary measure to inactivate as much myrosinase enzyme as possible before liquid extraction. 1 ml of preheated 70% (v/v) methanol (70 °C) was then added to each sample and placed in a water bath for 20 min at 70 °C. Samples were then centrifuged for 5 min (6000 rpm, 18 °C) to collect loose material into a pellet. The supernatant was then taken and put into fresh Eppendorf tubes. The volume was adjusted to 1 ml with 70% (v/v) methanol and frozen at −80 °C until analysis by LC–MS.

### 2.4 LC–MS^2^ analysis

Immediately before LC–MS analysis each sample was filtered using 0.25 μm filter discs with a low protein binding Durapore polyvinylidene fluoride (PVDF) membrane (Millex; EMD Millipore, Billerica, MA, USA) and diluted with 9 ml of HPLC-grade water. Samples were run in a random order with QC samples (Dunn, Wilson, Nicholls, & Broadhurst, 2012). An external reference standard of sinigrin hydrate was also prepared for quantification of GSL compounds, and isorhamnetin for flavonol compounds. Preparation was as follows: A 12 mM solution was prepared in 70% methanol. A dilution series of concentrations was prepared as an external calibration curve with HPLC-grade water (200, 150, 100, 56, 42, 28, 14 and 5.6 ng μl; sinigrin correlation coefficient: *y *= 12.496*x* − 15.012; *r*^2^ = 0.993, isorhamnetin correlation coefficient: *y = *0.3205*x* − 5.3833, *r*^2^ = 0.921). Standard response factors were used in the calculation of GSL concentration where available (Wathelet, Iori, Leoni, Quinsac, & Palmieri, 2004). Where such data could not be found for intact GSLs, response factors were assumed to be 1.00 (Lewis & Fenwick, 1987).

LC–MS analysis was performed in the negative ion mode on an Agilent 1200 Series LC system equipped with a binary pump, degasser, autosampler, thermostat, column heater, photodiode array detector and Agilent 1100 Series LC/MSD mass trap spectrometer. Separation of samples was achieved on a Zorbax SB C18 column (2.1 × 100 mm; 1.8 μm; Agilent, Santa Clara, CA, USA) with precolumn filter. Both GSLs and flavonols were separated in the same sample during a 40-min chromatographic run. Mobile phases consisted of ammonium formate (0.1%) and acetonitrile with a gradient of 95% and 5% respectively at a flow rate of 0.3 ml/min, with a column temperature of 30 °C. 5 μl of sample was injected.

MS analysis settings were as follows: ESI was carried out at atmospheric pressure in negative ion mode (scan range *m/z* 50–1050 Da). Nebulizer pressure was set at 50psi, gas-drying temperature at 350 °C, and capillary voltage at 20,000 V. Compounds were identified using their nominal mass and characteristic fragment ions, and by comparing data with those published in the literature (see Table 1, Table 2). GSLs were quantified at a wavelength of 229 nm, and flavonols at 330 nm. All data were analysed using Bruker Daltronics software.

**Table 1:** Identification of intact glucosinolates of *Eruca* and *Diplotaxis* varieties and accessions.

Common name	R-group	[M−H]^−^*m/z*	MS^2^	References
4-Hydroxyglucobrassicin	4-Hydroxy-3-indolylmethyl	463	381	Pasini et al., 2012, Rochfort et al., 2008
Glucotropaeolin	Benzyl	408	328, 259, 195	D’Antuono et al., 2008, Rochfort et al., 2008
Glucolepiidin	Ethyl	346	266	D’Antuono et al. (2008)
Glucoraphanin	4-(Methylsulfinyl)-butyl	436	371, 194	Bennett et al., 2002, Botting et al., 2002, Pasini et al., 2012, Rochfort et al., 2008
Glucoiberverin	3-(Methylthio)-propyl	406	325, 274, 258, 227	Fahey et al., 2001, Rochfort et al., 2008
Glucosativin	4-Mercaptobutyl	406	258, 209, 194, 138	Bennett et al., 2002, Lelario et al., 2012, Pasini et al., 2012
DMB	Dimeric-4-mercaptobutyl	811	731, 405, 258, 207	
Glucoalyssin	5-(Methylsulfinyl)-pentyl	450	371	Lelario et al., 2012, Pasini et al., 2012
Glucoerucin	4-(Methylthio)-butyl	420	339, 274, 258, 241, 194	Pasini et al., 2012, Rochfort et al., 2008
Glucoraphenin	4-Methylsulfinyl-3-butenyl	434	354	D’Antuono et al. (2008)
Diglucothiobeinin	4-(β-d-Glucopyranosyldisulfanyl)-butyl	600	–	Lelario et al., 2012, Pasini et al., 2012
Glucoibarin	7-(Methylsulfinyl)-heptyl	494	415	D’Antuono et al. (2008)

**Table 2:** Identification of flavonol of *Eruca* and *Diplotaxis* varieties and accessions.

Common name	[M−H]^−^*m/z*	MS^2^	References
Myricetin	317	151	Villatoro-Pulido et al. (2013)
Kaempferol-3-glucoside (Astragalin)	447	285	Martinez-Sanchez et al., 2008, Pasini et al., 2012
Quercetin-3-glucoside (Isoquercetrin)	463	301	
Isorhamnetin-3-glucoside	477	357, 314, 285, 151	
Kaempferol-3,4′-diglucoside	609	447, 285	
Isorhamnetin-3,4′-diglucoside	639	477	
Kaempferol-3-diglucoside-7-glucoside	771	609	Pasini et al. (2012)
Quercetin-3,3,4′-triglucoside	787	625, 463, 301	Martinez-Sanchez et al., 2008, Martinez-Sanchez et al., 2007, Pasini et al., 2012
Kaempferol-3-(2-sinapoyl-glucoside)-4′-glucoside	817	729, 685, 653, 447, 285	Martinez-Sanchez et al., 2008, Pasini et al., 2012
Quercetin-3,4′-diglucoside-3′-(6-caffeoyl-glucoside)	949	787, 625, 463, 301	Pasini et al. (2012)
Quercetin-3,4′-diglucoside-3′-(6-sinapoyl-glucoside)	993	831, 669, 463, 301	Martinez-Sanchez et al., 2008, Martinez-Sanchez et al., 2007, Pasini et al., 2012

### 2.5 Statistical analysis

The results reported are the averages of three biological replicates and three separately extracted technical replicates (*n *= 9). Processed GSL and flavonol data were analysed with ANOVA and Tukey’s HSD test, and principal component analysis (PCA) was performed in XL Stat (Addinsoft, New York City, New York, USA).

## 3 Results and discussions

### 3.1 Glucosinolate identification and concentration

Table 1 lists all of the GSL compounds identified across all rocket samples, including systematic names, common names and the identifying ions. Unlike previous studies, the GSL profiles of each rocket accession were markedly different in some cases. See Table 3 for a comparison of results with similar, previous studies. Total average GSL concentration ranged from 3.1 mg g^−1^ DW (Buzz) to 11.6 mg g^−1^ DW (SR10). Both of these accessions are *E.* *sativa*, indicating the large degree of variability between accessions of this species, both commercial and germplasm. The lowest average accumulation for *Diplotaxis* was Wild Tirizia with 4.4 mg g^−1^ DW and the highest was 10.4 mg g^−1^ DW, (Wild Grazia).

**Table 3:** Concentration ranges reported in mg g^−1^ DW (conversion of μmol g^−1^ DW of sinigrin hydrate) and days growth after sowing when plants were harvested.

Glucosinolate	Bennett et al. (2007)*ce*	Chun et al. (2013)*h*	Jin et al. (2009)*ce*	Kim & Ishii (2006)*h*	Pasini et al. (2012)*f*	Villatoro-Pulido et al. (2013)*f*	This study *ce*
	∼7 days	69 days	56 days	49 days	?	>56 days	30 days
Glucoerucin	0.0–12.7	0.3–2.2	0.0– ∼1.5	1.3	0.2–0.5	0.1–1.8	0.0–1.6
Glucoraphanin	0.2–2.7	0.4–1.7	0.0– ∼1.0	0.5	0.2–1.3	1.6–6.5	0.0–0.9
Glucoiberverin	ND	ND	0.0– ∼2.0	ND	ND	0.1–0.3	0.0–0.1
Glucosativin	0.2–14.5	ND	2.0– ∼7.0	ND	ND	3.2–4.6	0.2–9.1
4-Hydroxyglucobrassicin	ND	ND	ND	ND	<0.1–0.1	0.1	0.0–0.1
Diglucothiobeinin	ND	ND	0.0– ∼0.5	0.3	0.1	ND	0.0–0.2
DMB	ND	1.5–7.7	ND	2.3	0.2–0.7	ND	0.0–7.1
Glucoalyssin	ND	ND	ND	ND	<0.1–0.1	ND	0.0–0.1

ND = not detected, *ce *= controlled environment, *f = *field environment, *h = *hydroponic environment.

? represents an unknown value.

For glucosativin both the monomeric and the dimeric forms were identified and quantified separately and concentrations of both forms varied significantly between accessions. On average 91.3% of the total GSL concentration was made up of glucosativin/DMB. This is much higher than the proportions presented in previous studies where values of around 60% have been generally given (Pasini, Verardo, Caboni, & D’Antuono, 2012).

Other GSL compounds such as glucoraphanin and glucoerucin were not detected in all accessions. Again, previous studies have highlighted the prevalence of these compounds, but we found them to be relatively minor. Concentrations ranged from nil to 0.9 mg g^−1^ DW (Wild Grazia) for glucoraphanin and nil to 1.6 mg g^−1^ DW (SR16) for glucoerucin. Several other GSLs were quantified, and in some cases these were as high as the more generally accepted ‘major’ GSLs of rocket in concentration. The other compounds were: 4-hydroxyglucobrassicin, glucotropaeolin, glucolepiidin, glucoiberverin, glucoalyssin, glucoraphenin, diglucothiobeinin and glucoibarin. None of these GSLs discriminated between species.

In general, the concentrations detected were similar to those found in other studies. In some of these, plants were grown in field conditions and therefore subject to many different environmental stresses and inconsistencies. It is widely known that both GSLs and flavonols increase in concentration as plants become stressed (Rohr, Ulrichs, Mucha-Pelzer, & Mewis, 2006). With this in mind it is somewhat unusual that the concentrations reported here were not lower, as stress was minimal in comparison to field conditions. Studies conducted in outdoor conditions are not directly comparable for this reason. Field conditions and climate vary greatly between growing regions and GSL proportions may change due to these variables. Our study represents GSL and flavonol accumulation in rocket varieties and species under conditions that can be easily replicated using controlled environment apparatus. This allows the basic genetic differences in GSL profile to be observed, rather than the differences between how accessions respond to their normal, field-based growing environment. A trial of five gene bank accessions used in this study have been grown under field conditions and will be analysed using identical LC/MS methods to determine the effects the outdoor environment has on GSL and flavonol profiles.

Table 3 summarizes the range of concentrations of some GSLs previously reported in comparison with our own data. The types of growing method employed vary, as do the number of days growth before harvest. This makes comparing and contrasting between studies difficult and could potentially lead to erroneous conclusions. The details of these varying factors are discussed in Section 4.

### 3.2 Flavonol identification and concentration

Table 2 lists all identified flavonol compounds detected across all samples, including systematic names and identifying ions. In total eleven flavonol compounds were positively identified.

Myricetin was detected in relatively few accessions, but predominantly in *Eruca*. Previously this flavonol has not been identified in *Diplotaxis* species (to the authors’ knowledge), however, in this study it was detected in the commercial variety Wild Grazia.

Kaempferol glucosides kaempferol-3-glucoside (Astragalin) and kaempferol-3-diglucoside-7-glucoside have only been previously reported in *Eruca* species, but were additionally detected in two *Diplotaxis* varieties in our study (Wild Grazia and WR2). The ion fragments present in Table 2 confirmed their presence in these two commercial varieties. Kaempferol-3,4′-diglucoside was detected in both genera as reported by Pasini et al. (2012) and Martinez-Sanchez, Llorach, Gil, Ferreres, and Martínez-Sanchez (2007). The only kaempferol glucoside that was exclusive to *Eruca* species was kaempferol-3-(2-sinapoyl-glucoside)-4′-glucoside.

A similar situation was observed for quercetin glucosides. Quercetin-3-glucoside (Isoquercetrin) has only been previously reported in *Eruca* species, however it was also detected in one commercial accession of *Diplotaxis* (Wild Grazia). The converse was also found with quercetin-3,3,4′-triglucoside, quercetin-3,4′diglucoside-3′-(6-caffeoyl-glucoside) and quercetin-3,4′diglucoside-3′-(6-sinapoyl-glucoside), which have only previously been reported in *Diplotaxis*. These were detected in several *Eruca* accessions, as well as in *Diplotaxis*. Quercetin-3,3,4′-triglucoside showed the correct *m/z* 787 mass and secondary ions, and quercetin-3,4′diglucoside-3′-(6-caffeoyl-glucoside) was determined by the presence of a characteristic 625 fragment. Quercetin-3,4′-diglucoside-3′-(6-sinapoyl-glucoside) was determined by primary *m/z* 993 ion and corresponding secondary fragment ions (Table 2).

Two isorhamnetin glucosides were detected in our analysis; isorhamnetin-3-glucoside and isorhamnetin-3,4′-diglucoside. The latter compound was detected in both *Eruca* and *Diplotaxis* accessions, as has been reported in other studies (Martinez-Sanchez, Gil-Izquierdo, Gil, & Ferreres, 2008). Isorhamnetin-3-glucoside has only been previously reported in *Eruca*, but was also detected in seven *Diplotaxis* accessions (see Table 4).

**Table 4:** Total GSL concentration and relative amounts of each compound (± standard error) in rocket accessions (*n *= 9). Differing letters in the same column indicate a significant difference (*P = ⩽*0.05). Italics denote commercial varieties. Results are expressed as mg g^−1^ DW of sinigrin hydrate.

Accession name	Source	Species	4-Hydroxyglucobrassicin	Glucotropaeolin	Glucolepiidin	Glucoraphanin	Glucoiberverin	Glucosativin	DMB	Glucoalyssin	Glucoerucin	Glucoraphenin	Diglucothiobeinin	Glucoibarin	Average total GSLs (mg g^−1^ DW)
Apollo	Fothergills	Es	ND^a^	ND^a^	ND^a^	ND^a^	ND^a^	0.5 ± 0.2^a^	2.9 ± 0.7^a–e^	<0.1 ± <0.1^a^	ND^a^	0.1 ± 0.1^a^	ND^a^	ND^a^	3.6 ± 0.7^a^
Buzz	Fothergills	Es	ND^a^	ND^a^	ND^a^	ND^a^	ND^a^	1.3 ± 0.4^ab^	1.7 ± 0.3^a–c^	ND^a^	0.2 ± 0.2^a^	ND^a^	ND^a^	ND^a^	3.1 ± 0.6^a^
SR1	CGN	Es	ND^a^	ND^a^	ND^a^	0.5 ± 0.1^a^	ND^a^	6.0 ± 0.5^d–g^	ND^a^	ND^a^	ND^a^	ND^a^	<0.1 ± <0.1^a^	ND^a^	6.5 ± 0.5^ab^
SR2	CGN	Es	ND^a^	ND^a^	ND^a^	0.4 ± 0.1^a^	<0.1 ± <0.1^a^	3.5 ± 0.5^a–e^	2.3 ± 0.4^a–d^	ND^a^	0.3 ± 0.1^a^	<0.1 ± <0.1^a^	ND^a^	0.1 ± 0.1^ab^	6.6 ± 0.6^ab^
SR3	Elsoms Seeds Ltd.	Es	ND^a^	0.1 ± 0.1^a–c^	ND^a^	0.3 ± 0.1^a^	<0.1 ± <0.1^a^	2.7 ± 0.2^a–e^	1.2 ± 0.3^ab^	ND^a^	0.6 ± 0.2^ab^	ND^a^	ND^a^	ND^a^	4.9 ± 0.6^a^
SR4	IPK	Es	ND^a^	ND^a^	ND^a^	0.3 ± 0.1^a^	ND^a^	6.4 ± 1.3^e–g^	ND^a^	ND^a^	ND^a^	ND^a^	<0.1 ± <0.1^a^	ND^a^	6.7 ± 1.4^a–c^
SR5	IPK	Es	0.1 ± 0.1^a^	ND^a^	ND^a^	0.2 ± 0.1^a^	ND^a^	7.7 ± 0.8^fg^	3.3 ± 0.3^b–f^	<0.1 ± <0.1^a^	0.1 ± 0.1^a^	NDa	ND^a^	<0.1 ± <0.1^ab^	11.5 ± 0.9^c^
SR6	IPK	Es	ND^a^	ND^a^	ND^a^	0.6 ± 0.4^a^	0.1 ± <0.1^a^	3.5 ± 0.2^a–e^	4.4 ± 0.4^d–g^	<0.1 ± <0.1^a^	1.3 ± 0.3^ab^	0.2 ± 0.2^a^	ND^a^	ND^a^	10.0 ± 1.1^bc^
SR7	IPK	Es	ND^a^	ND^a^	ND^a^	0.3 ± 0.2^a^	ND^a^	4.8 ± 0.6^b–f^	2.7 ± 0.6^a–e^	ND^a^	0.1 ± 0.1^a^	ND^a^	ND^a^	ND^a^	7.9 ± 1.0^a–c^
SR8	IPK	Es	ND^a^	ND^a^	ND^a^	0.1 ± 0.1^a^	ND^a^	3.3 ± 1.1^a–e^	1.7 ± 0.4^a–c^	ND^a^	0.2 ± 0.2a	ND^a^	ND^a^	ND^a^	5.3 ± 1.8^ab^
SR9	IPK	Es	ND^a^	ND^a^	ND^a^	0.6 ± 0.3^a^	ND^a^	5.8 ± 0.7^d–g^	2.5 ± 1.0^a–e^	ND^a^	ND^a^	ND^a^	ND^a^	ND^a^	8.9 ± 1.0^a–c^
SR10	IPK	Es	ND^a^	ND^a^	ND^a^	0.4 ± 0.3^a^	ND^a^	9.1 ± 1.8^g^	1.4 ± 0.6^a–c^	ND^a^	0.7 ± 0.4^ab^	ND^a^	ND^a^	ND^a^	11.6 ± 2.1^c^
SR11	IPK	Es	ND^a^	ND^a^	ND^a^	ND^a^	ND^a^	5.4 ± 0.6^c–g^	0.1 ± 0.1^a^	NDa	ND^a^	ND^a^	ND^a^	ND^a^	5.6 ± 0.7^ab^
SR12	IPK	Es	ND^a^	ND^a^	ND^a^	0.3 ± 0.2^a^	0.1 ± <0.1^a^	5.7 ± 0.7^d–g^	1.9 ± 0.4^a–c^	ND^a^	0.5 ± 0.3^a^	ND^a^	ND^a^	ND^a^	8.4 ± 0.8^a–c^
SR13	IPK	Es	ND^a^	ND^a^	ND^a^	ND^a^	ND^a^	5.1 ± 0.5^b–f^	3.1 ± 0.6^a–f^	ND^a^	ND^a^	ND^a^	ND^a^	ND^a^	8.2 ± 0.6^a–c^
SR14	IPK	Es	ND^a^	<0.1 ± <0.1^ab^	ND^a^	ND^a^	<0.1 ± <0.1^a^	5.1 ± 0.4^c–f^	2.2 ± 0.5^a–d^	<0.1 ± <0.1^a^	0.1 ± 0.1^a^	ND^a^	ND^a^	ND^a^	7.5 ± 0.7^a–c^
SR15	IPK	Es	ND^a^	ND^a^	ND^a^	ND^a^	ND^a^	3.2 ± 1.1^a–e^	2.5 ± 0.5^a–e^	ND^a^	ND^a^	ND^a^	ND^a^	ND^a^	5.7 ± 1.5^ab^
SR16	IPK	Es	ND^a^	ND^a^	ND^a^	0.4 ± 0.1^a^	ND^a^	6.0 ± 0.9^d–g^	0.7 ± 0.3^ab^	ND^a^	1.6 ± 0.7^b^	ND^a^	ND^a^	ND^a^	8.7 ± 1.2^a–c^
SR17	IPK	Es	ND^a^	ND^a^	ND^a^	0.8 ± 0.1^a^	ND^a^	3.6 ± 0.5^a–e^	2.4 ± 0.6^a–e^	ND^a^	0.5 ± 0.1^ab^	ND^a^	ND^a^	ND^a^	7.3 ± 0.9^a–c^
SR18	IPK	Es	ND^a^	ND^a^	ND^a^	0.4 ± 0.2^a^	ND^a^	5.8 ± 1.1^d–g^	ND^a^	ND^a^	0.2 ± 0.2^a^	ND^a^	ND^a^	ND^a^	6.4 ± 1.3^ab^
SR19	IPK	Es	0.1 ± 0.1^a^	ND^a^	ND^a^	0.3 ± 0.1^a^	ND^a^	3.4 ± 0.5^a–e^	2.2 ± 0.6^a–d^	0.1 ± 0.1^a^	ND^a^	ND^a^	ND^a^	ND^a^	6.3 ± 0.8^ab^
SR20	IPK	Es	ND^a^	ND^a^	ND^a^	0.2 ± 0.1^a^	ND^a^	4.3 ± 1.4^a–f^	ND^a^	ND^a^	ND^a^	ND^a^	0.2 ± 0.1^b^	ND^a^	4.5 ± 1.4^a^
Pegasus	Tozer Seed	Es	ND^a^	ND^a^	ND^a^	ND^a^	ND^a^	0.7 ± 0.5^a^	3.1 ± 0.9^a–f^	ND^a^	ND^a^	ND^a^	ND^a^	0.1 ± 0.1^ab^	3.9 ± 1.3^a^
Runway	Fothergills	Es	ND^a^	ND^a^	0.1 ± 0.1^a^	ND^a^	ND^a^	0.2 ± 0.2^a^	3.3 ± 0.5^b–f^	0.1 ± 0.1^a^	ND^a^	ND^a^	ND^a^	ND^a^	3.6 ± 0.5^a^
Sky	Tozer Seed	Es	ND^a^	ND^a^	ND^a^	ND^a^	ND^a^	2.0 ± 0.5^a–d^	3.7 ± 0.5^b–g^	ND^a^	ND^a^	ND^a^	ND^a^	ND^a^	5.7 ± 0.9^ab^
Sweet oakleaf	Tozer Seed	Ev	ND^a^	ND^a^	ND^a^	ND^a^	ND^a^	1.0 ± 0.4^ab^	2.0 ± 0.7^a–d^	ND^a^	ND^a^	ND^a^	ND^a^	0.1 ± 0.1^ab^	3.3 ± 1.0^a^
Unwins	Unwins	Ev	ND^a^	ND^a^	ND^a^	0.1 ± 0.1^a^	ND^a^	1.6 ± 0.7^a–c^	7.1 ± 1.3^g^	0.1 ± 0.1^a^	ND^a^	<0.1 ± <0.1^a^	ND^a^	0.1 ± 0.1^ab^	9.0 ± 1.9^a–c^
Unwins organic	Unwins	Ev	ND^a^	ND^a^	0.1 ± 0.1^b^	0.1 ± 0.1^a^	ND^a^	0.9 ± 0.2^a–c^	4.3 ± 0.5^c–g^	<0.1 ± <0.1^a^	0.4 ± 0.2^a^	0.1 ± 0.1^a^	ND^a^	0.1 ± 0.1^ab^	5.9 ± 0.6^ab^
Olivetta	Fothergills	Dt	ND^a^	0.2 ± 0.2^bc^	ND^a^	ND^a^	ND^a^	1.4 ± 0.7^a–c^	4.9 ± 1.1^d–g^	ND^a^	ND^a^	ND^a^	ND^a^	ND^a^	6.6 ± 1.6^ab^
WR1	Bakkavor	Dt	0.1 ± 0.1^a^	ND^a^	ND^a^	ND^a^	ND^a^	1.7 ± 0.7^a–c^	3.8 ± 0.9^b–g^	ND^a^	ND^a^	<0.1 ± <0.1^a^	ND^a^	0.3 ± 0.1^b^	5.9 ± 1.5^ab^
Voyager	Tozer Seed	Dt	ND^a^	ND^a^	<0.1 ± <0.1^a^	<0.1 ± <0.1^a^	0.1 ± 0.1^a^	4.8 ± 0.7^b–f^	3.2 ± 0.5^b–f^	ND^a^	0.1 ± 0.1^a^	ND^a^	ND^a^	0.2 ± 0.1^b^	8.5 ± 0.5^a–c^
Wild Grazia	Fothergills	Dt	ND^a^	0.1 ± 0.1^a–c^	ND^a^	0.9 ± 0.6^a^	ND^a^	3.6 ± 0.7^a–e^	5.6 ± 1.0^e–g^	ND^a^	0.3 ± 0.3^a^	ND^a^	ND^a^	ND^a^	10.4 ± 2.1^bc^
Wild Tirizia	Fothergills	Dt	ND^a^	ND^a^	ND^a^	0.3 ± 0.2^a^	ND^a^	1.4 ± 0.5^a–c^	2.6 ± 0.4^a–e^	ND^a^	ND^a^	ND^a^	ND^a^	0.1 ± 0.1^ab^	4.4 ± 0.8^a^
Wildfire	Tozer Seed	Dt	ND^a^	0.3 ± 0.2^c^	ND^a^	ND^a^	ND^a^	1.6 ± 0.8^a–c^	7.0 ± 0.9^fg^	ND^a^	ND^a^	ND^a^	ND^a^	0.1 ± 0.1^ab^	9.0 ± 1.6^a–c^
WR2	Bakkavor	Dt	ND^a^	ND^a^	0.2 ± 0.2^b^	ND^a^	ND^a^	2.6 ± 1.3^a–e^	5.5 ± 1.4^d–g^	ND^a^	0.8 ± 0.7^ab^	ND^a^	ND^a^	0.2 ± 0.1^b^	9.3 ± 3.3^a–c^

ND = not detected, Es = *Eruca sativa,* Ev = *Eruca vesicaria,* Dt = *Diplotaxis tenuifolia.*

The concentration of each identified flavonol glucoside is presented in Table 5. As a general, overall observation, it can be said that *Diplotaxis* accessions have greater concentrations of quercetin flavonol compounds than *Eruca*, and the converse could be said for kaempferol. However using this as a broad, sweeping view to classify the two genera would be a mistake. Our results clearly show the cross genera presence of substantial concentrations of different flavonols that are by no means exclusive to one or the other. Indeed the two species may still be in the process of evolutionary divergence as far as phytochemical content is concerned. Total average flavonol content ranged from 0.5 g kg^−1^ DW (SR7) to 3.8 g kg^−1^ DW (Unwins) in *Eruca* samples, and from 0.6 g kg^−1^ DW (WR1) to 1.6 g kg^−1^ DW (Wild Grazia) in *Diplotaxis*.

**Table 5:** Total flavonol concentration and relative amounts of each compound (± standard error) in rocket accessions (*n *= 9). Differing letters in the same column indicate a significant difference (*P = *⩽0.05). Italics denote commercial varieties. Results are expressed as g kg^−1^ DW of isorhamnetin.

Accession name	Myricetin	Kaempferol-3-glucoside	Quercetin-3-glucoside	Isorhamnetin-3-glucoside	Kaempferol-3,4′-diglucoside	Isorhamnetin-3,4′-diglucoside	Kaempferol-3-diglucoside-7-glucoside	Quercetin-3,3,4′-triglucoside	Kaempferol-3-(2-sinapoyl-glucoside)-4′-glucoside	Quercetin-3,4′diglucoside-3′-(6-caffeoyl-glucoside)	Quercetin-3,4′diglucoside-3′-(6-sinapoyl-glucoside)	Average total flavonol (g.kg^−1^ DW)
Apollo	<0.1 ± <0.1^ab^	0.4 ± 0.1^a–c^	ND^a^	<0.1 ± <0.1^ab^	0.6 ± 0.1^a–d^	0.2 ± 0.1^a^	0.4 ± 0.1^ab^	ND^a^	0.4 ± 0.1^a–c^	0.1 ± 0.1^ab^	ND^a^	2.5 ± 0.4^a–c^
Buzz	<0.1 ± <0.1^a–c^	0.2 ± 0.1^ab^	0.1 ± 0.1^a^	0.2 ± 0.1^ab^	0.5 ± 0.1^a–d^	0.5 ± 0.1^a–c^	0.3 ± 0.1^ab^	0.1 ± 0.1^a^	ND^a^	0.5 ± 0.1^ab^	ND^a^	2.6 ± 0.3^bc^
SR1	ND^a^	0.1 ± <0.1^a^	ND^a^	0.1 ± <0.1^ab^	0.4 ± 0.1^a–c^	0.2 ± 0.1^ab^	ND^a^	<0.1 ± <0.1^a^	ND^a^	ND^a^	ND^a^	0.9 ± 0.2^ab^
SR2	<0.1 ± <0.1^ab^	0.1 ± <0.1^a^	<0.1 ± <0.1^a^	0.1 ± <0.1^ab^	0.5 ± 0.1^a–c^	0.5 ± 0.1^a–c^	0.2 ± 0.1^ab^	0.2 ± 0.1^ab^	0.2 ± 0.1^ab^	0.4 ± 0.1^ab^	ND^a^	2.5 ± 0.5^bc^
SR3	<0.1 ± <0.1^ab^	0.1 ± <0.1^a^	ND^a^	0.1 ± <0.1^ab^	0.4 ± 0.1^a–c^	0.2 ± 0.1^a^	0.1 ± <0.1^a^	0.1 ± <0.1^a^	0.1 ± 0.1^ab^	0.2 ± 0.1^ab^	ND^a^	1.3 ± 0.2^ab^
SR4	ND^a^	0.2 ± 0.1^a^	<0.1 ± <0.1^a^	0.2 ± <0.1^ab^	0.5 ± 0.2^a–c^	0.3 ± 0.2^ab^	ND^a^	ND^a^	ND^a^	ND^a^	ND^a^	1.2 ± 0.4^ab^
SR5	ND^a^	0.3 ± <0.1^ab^	ND^a^	ND^a^	0.5 ± 0.1^a–d^	0.4 ± 0.1^ab^	<0.1 ± <0.1^a^	<0.1 ± <0.1^a^	0.1 ± 0.1^ab^	0.3 ± 0.1^ab^	0.1 ± 0.1^ab^	1.9 ± 0.3^a–c^
SR6	ND^a^	0.4 ± 0.1^ab^	ND^a^	ND^a^	1.1 ± 0.1^e^	0.6 ± 0.1^bc^	0.2 ± 0.1^ab^	<0.1 ± <0.1^a^	0.1 ± <0.1^a^	0.4 ± 0.1^ab^	ND^a^	3.2 ± 0.4^c^
SR7	ND^a^	0.2 ± 0.1^a^	ND^a^	<0.1 ± <0.1^ab^	0.3 ± 0.1^a–c^	<0.1 ± <0.1^a^	ND^a^	ND^a^	ND^a^	ND^a^	ND^a^	0.5 ± 0.1^a^
SR8	ND^a^	0.8 ± 0.2^c^	ND^a^	0.2 ± 0.1^ab^	1.0 ± 0.1^de^	ND^a^	ND^a^	ND^a^	ND^a^	ND^a^	ND^a^	1.8 ± 0.3^a–c^
SR9	ND^a^	0.4 ± 0.1^ab^	<0.1 ± <0.1^a^	ND^a^	1.0 ± 0.3^de^	1.0 ± 0.4^c^	ND^a^	<0.1 ± <0.1^a^	<0.1 ± <0.1^a^	ND^a^	ND^a^	2.5 ± 0.7^a–c^
SR10	ND^a^	0.2 ± <0.1^a^	<0.1 ± <0.1^a^	0.2 ± 0.1^ab^	0.4 ± 0.1^a–c^	0.2 ± 0.1^a^	ND^a^	ND^a^	ND^a^	ND^a^	ND^a^	1.0 ± 0.2^ab^
SR11	ND^a^	0.1 ± <0.1^a^	ND^a^	<0.1 ± <0.1^ab^	0.4 ± <0.1^a–c^	0.2 ± 0.1^a^	ND^a^	ND^a^	ND^a^	ND^a^	ND^a^	0.7 ± 0.1^a^
SR12	ND^a^	0.2 ± 0.1^a^	ND^a^	0.1 ± <0.1^ab^	0.6 ± 0.1^a–d^	0.3 ± 0.1^ab^	0.1 ± 0.1^a^	0.1 ± 0.1^a^	0.2 ± 0.1^a–c^	0.3 ± 0.1^ab^	ND^a^	2.3 ± 0.5^a–c^
SR13	ND^a^	0.1 ± <0.1^a^	ND^a^	ND^a^	0.6 ± <0.1^a–d^	0.2 ± 0.1^a^	ND^a^	ND^a^	ND^a^	ND^a^	ND^a^	0.8 ± 0.1^ab^
SR14	ND^a^	0.3 ± 0.1^ab^	ND^a^	0.1 ± <0.1^ab^	0.6 ± 0.1^a–d^	0.1 ± 0.1^a^	0.1 ± 0.1^a^	ND^a^	0.1 ± 0.1^ab^	0.3 ± 0.1^ab^	ND^a^	1.7 ± 0.3^a–c^
SR15	ND^a^	0.2 ± <0.1^a^	ND^a^	ND^a^	0.7 ± 0.1^b–e^	<0.1 ± <0.1^a^	ND^a^	ND^a^	ND^a^	ND^a^	ND^a^	1.0 ± 0.1^ab^
SR16	ND^a^	0.1 ± <0.1a	<0.1 ± <0.1^a^	0.1 ± <0.1^ab^	0.3 ± 0.1^a–c^	0.2 ± 0.1^ab^	ND^a^	ND^a^	ND^a^	ND^a^	ND^a^	0.9 ± 0.2^ab^
SR17	ND^a^	0.1 ± <0.1^a^	ND^a^	ND^a^	0.4 ± 0.1^a–c^	0.2 ± 0.1^ab^	ND^a^	ND^a^	ND^a^	<0.1 ± <0.1^a^	ND^a^	0.7 ± 0.2^a^
SR18	ND^a^	0.2 ± 0.1^ab^	<0.1 ± <0.1^a^	0.2 ± <0.1^ab^	0.4 ± 0.2^a–c^	0.2 ± 0.2^ab^	ND^a^	<0.1 ± <0.1^a^	ND^a^	ND^a^	ND^a^	1.1 ± 0.4^ab^
SR19	ND^a^	0.2 ± 0.1^a^	ND^a^	ND^a^	0.3 ± 0.1^a–c^	0.4 ± 0.1^ab^	0.1 ± 0.1^a^	<0.1 ± <0.1^a^	<0.1 ± <0.1^a^	0.2 ± 0.1^ab^	ND^a^	1.4 ± 0.3^ab^
SR20	ND^a^	0.2 ± <0.1^a^	0.1 ± 0.1^a^	0.1 ± <0.1^ab^	0.4 ± 0.1^a–c^	0.3 ± 0.1^ab^	ND^a^	<0.1 ± <0.1^a^	ND^a^	ND^a^	ND^a^	1.1 ± 0.2^ab^
Pegasus	ND^a^	0.6 ± 0.3^bc^	ND^a^	0.4 ± 0.3^a–c^	0.3 ± 0.1^a–c^	<0.1 ± <0.1^a^	0.5 ± 0.3^b^	ND^a^	0.2 ± <0.1^a–c^	0.2 ± 0.1^ab^	ND^a^	2.7 ± 1.0^bc^
Runway	<0.1 ± <0.1^ab^	0.3 ± 0.1^ab^	ND^a^	0.4 ± 0.1^a–c^	0.6 ± 0.2^a–d^	0.6 ± 0.1^a–c^	0.2 ± 0.1^ab^	<0.1 ± <0.1^a^	0.1 ± 0.1^ab^	0.6 ± 0.2^b^	0.3 ± 0.1^b–d^	3.1 ± 1.0^bc^
Sky	0.1 ± <0.1^c^	0.2 ± <0.1^ab^	ND^a^	0.1 ± 0.1^ab^	0.6 ± 0.1^a–d^	0.4 ± 0.1^a–c^	0.2 ± 0.1^ab^	0.4 ± 0.2^b^	0.3 ± 0.2^a–c^	0.2 ± 0.1^ab^	ND^a^	2.6 ± 0.5^bc^
Sweet oakleaf	0.2 ± 0.1^c^	0.2 ± 0.1^ab^	<0.1 ± <0.1^a^	<0.1 ± <0.1^ab^	0.4 ± <0.1^a–c^	0.3 ± 0.1^ab^	0.1 ± 0.1^a^	0.2 ± 0.1^ab^	ND^a^	0.4 ± 0.1^ab^	ND^a^	1.7 ± 0.2^a–c^
Unwins	<0.1 ± <0.1^bc^	0.5 ± 0.1^bc^	ND^a^	0.3 ± 0.1^ab^	0.8 ± 0.1^c–e^	0.2 ± 0.1^ab^	0.4 ± 0.1^ab^	ND^a^	0.6 ± 0.3^c^	0.6 ± 0.2^b^	ND^a^	3.8 ± 0.5^c^
Unwins Organic	<0.1 ± <0.1^ab^	0.3 ± 0.1^ab^	ND^a^	0.1 ± 0.1^ab^	0.6 ± 0.1^a–d^	<0.1 ± <0.1^a^	0.3 ± 0.1^ab^	ND^a^	0.4 ± 0.2^bc^	0.4 ± 0.1^ab^	0.1 ± 0.1^ab^	2.5 ± 0.2^a–c^
Olivetta	ND^a^	ND^a^	ND^a^	0.4 ± 0.3^a–c^	<0.1 ± <0.1^a^	0.4 ± 0.2^ab^	ND^a^	0.2 ± <0.1^ab^	ND^a^	ND^a^	0.1 ± 0.1^a–c^	0.9 ± 0.5^ab^
WR1	ND^a^	ND^a^	ND^a^	0.3 ± 0.2^ab^	ND^a^	0.1 ± <0.1^a^	ND^a^	0.2 ± <0.1^ab^	ND^a^	<0.1 ± <0.1^a^	0.4 ± 0.1^cd^	0.6 ± 0.2^a^
Voyager	ND^a^	ND^a^	ND^a^	0.3 ± 0.2^ab^	ND^a^	0.1 ± <0.1^a^	ND^a^	0.2 ± <0.1^ab^	ND^a^	<0.1 ± <0.1^a^	0.4 ± 0.1^d^	0.7 ± 0.4^a^
Wild Grazia	<0.1 ± <0.1^ab^	0.1 ± 0.1^a^	0.1 ± 0.1^a^	0.6 ± 0.2^bc^	0.1 ± 0.1^a^	0.2 ± 0.1^ab^	0.1 ± 0.1^a^	0.2 ± <0.1^ab^	ND^a^	0.1 ± 0.1^ab^	0.7 ± 0.1^e^	1.6 ± 0.6^a–c^
Wild Tirizia	ND^a^	ND^a^	ND^a^	0.4 ± 0.1^a–c^	ND^a^	0.1 ± <0.1^a^	ND^a^	0.2 ± <0.1^ab^	ND^a^	0.1 ± <0.1^a^	0.2 ± 0.1^a–d^	0.7 ± 0.1^ab^
Wildfire	ND^a^	ND^a^	ND^a^	1.0 ± 0.4^c^	ND^a^	0.1 ± <0.1^a^	ND^a^	0.2 ± <0.1^ab^	ND^a^	0.1 ± 0.1^ab^	0.1 ± 0.1^a–d^	1.5 ± 0.4^a–c^
WR2	ND^a^	0.1 ± 0.1^a^	ND^a^	0.4 ± 0.1^a–c^	0.2 ± 0.2^ab^	0.1 ± 0.1^a^	0.2 ± 0.2^ab^	0.1 ± <0.1^a^	ND^a^	0.1 ± <0.1^a^	0.4 ± 0.1^de^	1.1 ± 0.4^ab^

ND = not detected.

In agreement with Pasini et al. (2012) and Martinez-Sanchez et al. (2007), kaempferol-3,4′-diglucoside was the most common kaempferol glucoside detected. Isorhamnetin-3-glucoside concentrations ranged from nil to 1.0 g kg^−1^ DW (Wildfire), and isorhamnetin-3,4′-diglucoside similarly ranged from nil to 1.0 g kg^−1^ DW (SR10). Interestingly, flavonol concentrations were generally higher for commercial varieties than gene bank accessions. This may reflect inadvertent selection on the part of breeders when traits such as taste and flavour are considered.

Our results are roughly 20% of the concentrations that have been previously reported for rocket (Pasini et al., 2012). The controlled, unstressed growth environment used in our experiment may explain this. Jin et al. (2009) previously reported that flavonol concentrations are significantly affected by different light intensities. The outdoor equivalent to the light intensities used in our experiment would be akin to shade illuminated by an entire, clear blue sky at midday. Using this as a comparative scenario, the plants in this experiment experienced no direct sunlight stress conditions (equivalent to >2000 μmol m^−2^ s^−1^). Our method therefore offers a representation of unstressed conditions for rocket flavonol accumulation, as outdoor light intensities can vary greatly according to the growing region, climate and time of year.

### 3.3 Glucosinolate composition and profiles

The profiles of all rocket accessions tested were broadly similar in terms of composition. No GSLs were detected that discriminated between the different species or commercial/gene bank accessions, and the dominance of glucosativin and DMB on GSL content broadly rendered differentiation between samples difficult. PCA analyses (not presented) showed data extremely skewed in the direction of glucosativin. Although some accessions such as SR5 contained relatively rare (for rocket species) GSLs such as 4-hydroxyglucobrassicin and glucoibarin, these concentrations were not significant enough to discriminate on a PCA scores plot due to this dominance.

### 3.4 Flavonol composition and profiles

**Fig. 1:**
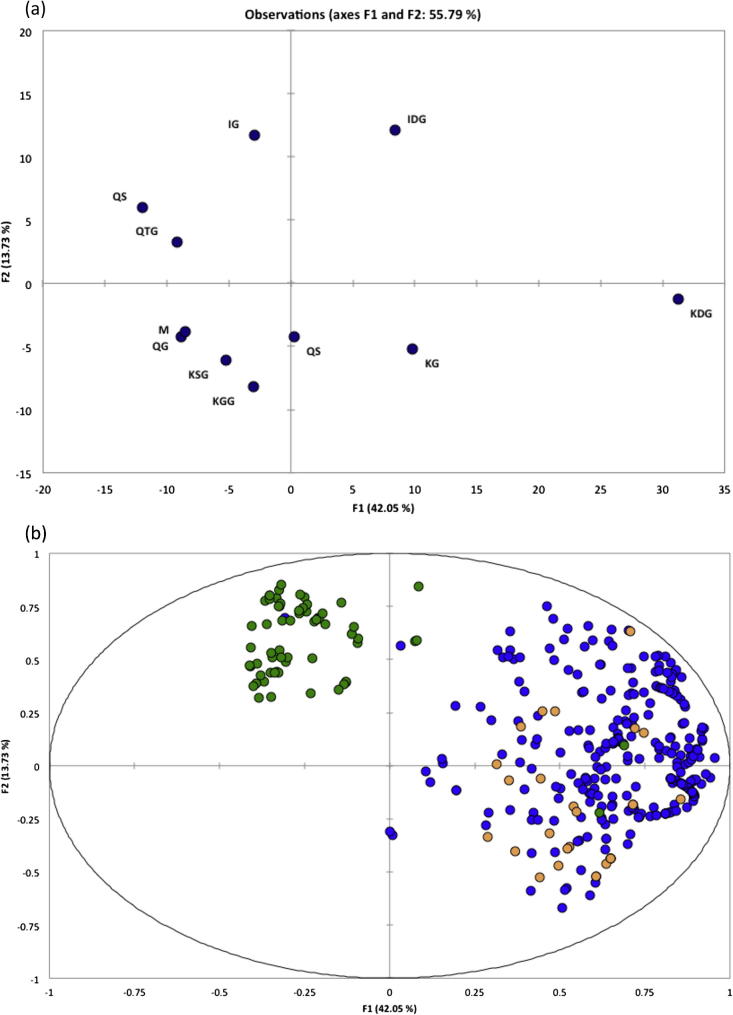
(a) PCA loadings plot of flavonol compounds detected by LC–MS analysis. Abbreviations: M, myricetin; KG, kaempferol-3-glucoside; QG, quercetin-3-glucoside; IG, isorhamnetin-3-glucoside; KDG, kaempferol-3,4′-diglucoside; IDG, isorhamnetin-3,4′-diglucoside; KGG, kaempferol-3-diglucoside-7-glucoside; QTG, quercetin-3,3,4′-triglucoside; KSG, kaempferol-3-(2-sinapoyl-glucoside)-4′-glucoside; QC, quercetin-3,4′-diglucoside-3′-(6-caffeoyl-glucoside); QS, quercetin-3,4′-diglucoside-3′-(6-sinapoyl-glucoside). (b) PCA scores plot for individual LC–MS samples tested and their relative distributions in relation to the loadings plot of flavonol composition. Green = *Diplotaxis tenuifolia*; Blue = *Eruca sativa*; Orange = *Eruca vesicaria*.

Flavonol composition was markedly different from GSL composition. Fig. 1 shows the scores and loadings plot of a PCA, where PCs 1 and 2 accounted for 55.79% of the observed variation. The scores plot shows a clear differentiation between *Diplotaxis* and *Eruca* with the two genera forming two distinct clusters. When compared with the loadings plot, it is clear that this divide is largely due to differences in kaempferol-3,4′-diglucoside and kaempferol-3-glucoside concentration in *Eruca*, and the tendency for *Diplotaxis* to accumulate quercetin and isorhamnetin glucosides in greater amounts.

## 4 Conclusions

### 4.1 Effects of growing conditions on GSL concentrations

This study has highlighted phytochemical accumulation for rocket varieties and accessions grown under controlled conditions. This is in contrast to field conditions that often stress plants and create phytochemical profiles reflective of fluctuating environmental stresses such as light intensity, temperature, pests and diseases. These studies, whilst undoubtedly valuable to rocket salad research, are not always directly comparable with other growing regions and climatic backgrounds. It has been demonstrated in this study that under controlled conditions, and therefore due to genetic regulation rather than environmental response, that rocket predominantly accumulates glucosativin, and that virtually all other glucosinolates detected were minor by comparison. There was significant variability in these accumulations between varieties, providing scope for plant breeders to select varieties based on their baseline accumulations of health-beneficial precursors such as glucoraphanin and glucoerucin. This can also be said of flavonol compounds detected in rocket. Significant variability was detected between accessions, and high accumulators may be a valuable genetic resource for breeders. By determining the baseline accumulations of phytochemicals in this manner, varieties can then be tested in a field environment to ascertain any differences that could affect commercial production.

### 4.2 Effects of time-of-harvest and plant maturity on GSL concentrations

Several previous studies have made mention of using phytochemical screening as a means of selecting accessions to introduce into breeding programs. In almost every instance however, the experimental design of these studies was flawed by the fact that time-of-harvest was either much too early or much too late relative to the commercial average. Not only does this make comparing results between studies more difficult, it also ignores the fact that phytochemical concentration and profiles change as plants grow (Fernandes, de Pinho, Valentao, Pereira, & Andrade, 2009). If researchers wish to make their data as useful to breeding programs as possible, the phytochemical profile must be determined at the point of commercial harvest, as this is when concentrations will be at their most useful in a “real-world” commercial setting. Plant breeders and food processors will not be interested in the phytochemical content of seedlings or of plants that have bolted or flowered (unless they provide products for a very niche market), as their customers will not eat the product at these points.

Table 3 features the number of days each of the mentioned studies grew rocket plants before harvesting. Regardless of growing conditions, the number generally chosen seems arbitrary. It is generally quoted within the literature that rocket is harvested anywhere between 30 and 60 days (Martínez-Sánchez, Allende, Cortes-Galera, Gil, & Martinez-Sanchez, 2008), however in reality it is more like between 25 and 35 days. Bolting and flowering in rocket varieties is highly variable, but in general, most will reach this stage before 45 days of growth. This is why in our study 30 days was chosen as the point of harvest, and was determined in consultation with commercial partners who grow rocket on a large scale, in the UK, Italy and Portugal.

Bennett, Carvalho, Mellon, Eagles, and Rosa (2007) harvested seedlings at the point where the cotyledons were fully expanded, which is typically around seven days of growth. This is not however the point at which growers will harvest their crop (unless it is marketed as a ‘microleaf’ product), and although GSL concentrations are likely to be higher in young leaves, this is not necessarily reflective of what the end consumer will receive. Conversely, the other studies all harvested at or after forty-nine days (with the exception of Pasini et al. (2012) where no point of harvest time was given). Whilst still theoretically within the commercial harvest window, it is unlikely that growers would wait this long to harvest a crop, as the demand for rocket is so high. Chun, Arasu, Lim, and Kim (2013) stated that their work was part of a breeding program to determine varieties with high concentrations of health promoting GSLs. However, the point of harvest was at 69 days, which is well beyond commercial viability. Indeed it is stated that plants were of a height of up to 46 cm when harvest occurred. From this it is clear that plants had begun flowering (or at the very least bolting), and as such, the GSL profile is likely to have altered substantially from the marketable stage of plant growth.

If researchers and breeders wish to effectively breed new varieties with enhanced phytochemical content, the consumer end-point and supply-chain must be considered in the experimental design. Selecting plants with high GSL concentrations at cotyledon and flowering stage will not necessarily be the same plants with the highest concentrations at the marketable stage.

### 4.3 Effects of genetics

Research into the underlying genetic mechanisms for GSL regulation has shown that MYB transcription factors are responsible. In *Arabidopsis thaliana* it has been shown that the HAG2/MYB76 and HAG3/MYB29 transcription factors are responsible for the biosynthesis of aliphatic GSLs and the down-regulation of indolic GSL biosynthesis (Gigolashvili, Engqvist, Yatusevich, Müller, & Flügge, 2008). This would seem to indicate that *Brassicaceae* plants are capable of adapting their GSL profile to different environmental stimuli. Very little specific research has been conducted in rocket in this regard, but it is likely that the species share analogous genes and transcription factors with both *A*. *thaliana* and *Brassica* crops. With detailed study into these mechanisms, it is possible that breeders could select plants based on sets of genes, to specify responses to different environments. In this way, health beneficial GSLs could be enhanced, and less desirable ones minimized or removed entirely. This could also apply to flavonols, which are also known to be regulated by MYB transcription factors (Stracke et al., 2007).

### 4.4 Commercial vs. Gene bank accessions

Our hypothesis that some phytochemical constituents have been lost through breeding does not appear to be wholly accurate. Whilst some gene bank accessions showed very high concentrations, others showed the exact opposite. The same can be said for the commercial varieties, as some were very poor accumulators of health beneficial compounds, but others contained high concentrations. It seems that whilst gene banks are a valuable resource for beneficial phytochemical traits, not all accessions are worth breeding from. Breeders must therefore screen as large a number of accessions as possible in order to pick out the very best material. The ‘super broccoli’ variety Beneforte was bred in a similar fashion to this, by utilising hybridization with wild relatives. Broccoli accumulates predominantly glucoraphanin within floret tissue, and through selective breeding a threefold increase in yield was achieved (variety 1639; ∼11.1 mg g^−1^ DW) (Traka et al., 2013). Although rocket does not contain such inherently high concentrations, being only a small plant by comparison, there is no reason why similar concerted efforts could not enhance accumulations of glucoraphanin or other GSLs for the purposes of benefitting the consumer. It also has the added benefit that it does not need to be cooked before eating. This eliminates myrosinase thermal degradation and maximizes the production of health-beneficial volatiles such as indoles and ITCs.

Both genera showed significant variation in terms of the overall presence and absence of different phytochemicals. Several flavonols have been detected in each species that have not been previously documented. This inherent variability between cultivars provides breeders and food producers with the opportunity to create products that are specific to the tastes and preferences of consumers. That being said, concentrations within accession groups and commercial varieties were highly variable in our study. More high quality breeding is needed to improve uniformity in this respect. The data produced in this study will be used actively in the production of new varieties of superior nutritional and sensory quality, in conjunction with industrial partners.

### 4.5 Future work

Despite the increase in rocket research in the last few years, much more study is needed to properly determine the effects of specific stresses on GSL composition and concentration. Here we have shown that concentrations under controlled conditions are generally in agreement with those of studies on field and hydroponic grown rocket. Flavonol concentration varied substantially however, and was likely due to controlled environment lighting conditions. Future work in our research group aims to compare field-grown material to the results presented here in order to properly determine which phytochemicals are affected by outdoor stresses, such as high light, high temperature, restricted water availability and increased growing density.

Researchers and breeders may need to consider more carefully the producer, supply chain, and end consumer when selecting material for breeding programs. Furthermore, much more work is needed to properly understand the degradation products of GSLs, and the underlying genetics responsible for which volatiles are produced by myrosinase interaction, in what proportions, and what effects this may have for human health.

## Funding

Luke Bell is supported by a 10.13039/501100000268BBSRC Case Award (Reference BB/J012629/1) in partnership with Elsoms Seeds Ltd. (Spalding, UK) and Bakkavor Group Ltd. (Spalding, UK).

## References

[b0005] Bennett R.N., Carvalho R., Mellon F.A., Eagles J., Rosa E.A.S. (2007). Identification and quantification of glucosinolates in sprouts derived from seeds of wild *Eruca sativa* L. (salad rocket) and *Diplotaxis tenuifolia* L. (wild rocket) from diverse geographical locations. Journal of Agricultural and Food Chemistry.

[b0010] Bennett R.N., Mellon F.A., Botting N.P., Eagles J., Rosa E.A.S., Williamson G. (2002). Identification of the major glucosinolate (4-mercaptobutyl glucosinolate) in leaves of *Eruca sativa* L. (salad rocket). Phytochemistry.

[b0015] Bennett R.N., Rosa E.A.S., Mellon F.A., Kroon P.A. (2006). Ontogenic profiling of glucosinolates, flavonoids, and other secondary metabolites in *Eruca sativa* (salad rocket), *Diplotaxis erucoides* (wall rocket), *Diplotaxis tenuifolia* (wild rocket), and *Bunias orientalis* (Turkish rocket). Journal of Agricultural and Food Chemistry.

[b0020] Bjorkman M., Klingen I., Birch A.N.E., Bones A.M., Bruce T.J.A., Johansen T.J. (2011). Phytochemicals of *Brassicaceae* in plant protection and human health – Influences of climate, environment and agronomic practice. Phytochemistry.

[b0025] Botting C.H., Davidson N.E., Griffiths D.W., Bennett R.N., Botting N.P. (2002). Analysis of intact glucosinolates by MALDI-TOF mass spectrometry. Journal of Agricultural and Food Chemistry.

[b0030] Bozokalfa M.K., Esiyok D., Yagmur B. (2011). Use of multivariate analysis in mineral accumulation of rocket (*Eruca sativa*) accessions. Genetika-Belgrade.

[b0035] Casagrande S.S., Wang Y., Anderson C., Gary T.L. (2007). Have Americans increased their fruit and vegetable intake? The trends between 1988 and 2002. American Journal of Preventive Medicine.

[b0040] Cataldi T.R.I., Rubino A., Lelario F., Bufo S.A. (2007). Naturally occurring glucosinolates in plant extracts of rocket salad (*Eruca sativa* L.) identified by liquid chromatography coupled with negative ion electrospray ionization and quadrupole ion-trap mass spectrometry. Rapid Communications in Mass Spectrometry.

[b0045] Chun J.-H., Arasu M.V., Lim Y.-P., Kim S.-J. (2013). Variation of major glucosinolates in different varieties and lines of rocket salad. Horticulture, Environment and Biotechnology.

[b0050] D’Antuono L.F., Elementi S., Neri R. (2008). Glucosinolates in *Diplotaxis* and *Eruca* leaves: Diversity, taxonomic relations and applied aspects. Phytochemistry.

[b0055] D’Antuono L.F., Elementi S., Neri R. (2009). Exploring new potential health-promoting vegetables: Glucosinolates and sensory attributes of rocket salads and related *Diplotaxis* and *Eruca* species. Journal of the Science of Food and Agriculture.

[b0060] Dunn W.B., Wilson I.D., Nicholls A.W., Broadhurst D. (2012). The importance of experimental design and QC samples in large-scale and MS-driven untargeted metabolomic studies of humans. Bioanalysis.

[b0065] Fahey J.W., Zalcmann A.T., Talalay P. (2001). The chemical diversity and distribution of glucosinolates and isothiocyanates among plants. Phytochemistry.

[b0070] Fernandes F., de Pinho P.G., Valentao P., Pereira J.A., Andrade P.B. (2009). Volatile constituents throughout *Brassica oleracea* L. Var. acephala germination. Journal of Agricultural and Food Chemistry.

[b0075] Gigolashvili T., Engqvist M., Yatusevich R., Müller C., Flügge U.-I. (2008). HAG2/MYB76 and HAG3/MYB29 exert a specific and coordinated control on the regulation of aliphatic glucosinolate biosynthesis in *Arabidopsis thaliana*. The New Phytologist.

[b0080] Jin J., Koroleva O.A., Gibson T., Swanston J., Magan J., Zhang Y. (2009). Analysis of phytochemical composition and chemoprotective capacity of rocket (*Eruca sativa* and *Diplotaxis tenuifolia*) leafy salad following cultivation in different environments. Journal of Agricultural and Food Chemistry.

[b0085] Kim S.J., Ishii G. (2006). Glucosinolate profiles in the seeds, leaves and roots of rocket salad (*Eruca sativa* Mill.) and anti-oxidative activities of intact plant powder and purified 4-methoxyglucobrassicin. Soil Science and Plant Nutrition.

[b0090] Kim S.J., Jin S., Ishii G. (2004). Isolation and structural elucidation of 4-(beta-d-glucopyranosyldisulfanyl)butyl glucosinolate from leaves of rocket salad (*Eruca sativa* L.) and its antioxidative activity. Bioscience, Biotechnology and Biochemistry.

[b0095] Kim S.J., Kawaharada C., Jin S., Hashimoto M., Ishii G., Yamauchi H. (2007). Structural elucidation of 4-(cysteine-S-yl)butyl glucosinolate from the leaves of *Eruca sativa*. Bioscience, Biotechnology and Biochemistry.

[b0100] Lelario F., Bianco G., Bufo S.A., Cataldi T.R.I. (2012). Establishing the occurrence of major and minor glucosinolates in *Brassicaceae* by LC-ESI-hybrid linear ion-trap and Fourier-transform ion cyclotron resonance mass spectrometry. Phytochemistry.

[b0105] Lewis J., Fenwick G.R. (1987). Glucosinolate content of brassica vegetables – Analysis of 24 cultivars of calabrese (green sprouting broccoli, *Brassica-Oleracea* L Var botrytis subvar cymosa lam). Food Chemistry.

[b0110] Lynn A., Collins A., Fuller Z., Hillman K., Ratcliffe B. (2006). Cruciferous vegetables and colo-rectal cancer. Proceedings of the Nutrition Society.

[b0115] Martínez-Sánchez A., Allende A., Cortes-Galera Y., Gil M.I., Martinez-Sanchez A. (2008). Respiration rate response of four baby leaf *Brassica* species to cutting at harvest and fresh-cut washing. Postharvest Biology and Technology.

[b0125] Martinez-Sanchez A., Gil-Izquierdo A., Gil M.I., Ferreres F. (2008). A comparative study of flavonoid compounds, vitamin C, and antioxidant properties of baby leaf *Brassicaceae* species. Journal of Agricultural and Food Chemistry.

[b0130] Martinez-Sanchez A., Llorach R., Gil M.I.M.I., Ferreres F., Martínez-Sanchez A. (2007). Identification of new flavonoid glycosides and flavonoid profiles to characterize rocket leafy salads (*Eruca vesicaria* and *Diplotaxis tenuifolia*). Journal of Agricultural and Food Chemistry.

[b0135] Martinez-Sanchez A., Marin A., Llorach R., Ferreres F., Gil M.I., Martínez-Sánchez A. (2006). Controlled atmosphere preserves quality and phytonutrients in wild rocket (*Diplotaxis tenuifolia*). Postharvest Biology and Technology.

[b0140] Pasini F., Verardo V., Caboni M.F., D’Antuono L.F. (2012). Determination of glucosinolates and phenolic compounds in rocket salad by HPLC-DAD–MS: Evaluation of *Eruca sativa* Mill. and *Diplotaxis tenuifolia* L. genetic resources. Food Chemistry.

[b0145] Pasini F., Verardo V., Cerretani L., Caboni M.F., D’Antuono L.F. (2011). Rocket salad (*Diplotaxis* and *Eruca* spp.) sensory analysis and relation with glucosinolate and phenolic content. Journal of the Science of Food and Agriculture.

[b0150] Rochfort S.J., Trenerry V.C., Imsic M., Panozzo J., Jones R. (2008). Class targeted metabolomics: ESI ion trap screening methods for glucosinolates based on MSn fragmentation. Phytochemistry.

[b0155] Rohr F., Ulrichs C., Mucha-Pelzer T., Mewis I. (2006). Variability of aliphatic glucosinolates in *Arabidopsis* and their influence on insect resistance. Communications in Agricultural and Applied Biological Sciences.

[b0160] Schranz M.E., Manzaneda A.J., Windsor A.J., Clauss M.J., Mitchell-Olds T. (2009). Ecological genomics of *Boechera stricta*: Identification of a QTL controlling the allocation of methionine- vs branched-chain amino acid-derived glucosinolates and levels of insect herbivory. Heredity.

[b0165] Stracke R., Ishihara H., Huep G., Barsch A., Mehrtens F., Niehaus K. (2007). Differential regulation of closely related R2R3-MYB transcription factors controls flavonol accumulation in different parts of the *Arabidopsis thaliana* seedling. The Plant Journal: For Cell and Molecular Biology.

[b0170] Traka M.H., Mithen R.F. (2011). Plant science and human nutrition: Challenges in assessing health-promoting properties of phytochemicals. Plant Cell.

[b0175] Traka M.H., Saha S., Huseby S., Kopriva S., Walley P.G., Barker G.C. (2013). Genetic regulation of glucoraphanin accumulation in Beneforté broccoli. The New Phytologist.

[b0180] Villatoro-Pulido M., Priego-Capote F., Alvarez-Snachez B., Saha S., Philo M., Obregon-Cano S. (2013). An approach to the phytochemical profiling of rocket [*Eruca sativa* (Mill.) Thell]. Journal of the Science of Food and Agriculture.

[b0185] Wathelet J.-P., Iori R., Leoni O., Quinsac A., Palmieri S. (2004). Guidelines for glucosinolate analysis in green tissues used for biofumigation. Agroindustria.

